# Modulation of the nociceptive withdrawal reflex in walking is not changed by the presence of tonic pain

**DOI:** 10.1007/s00221-026-07379-7

**Published:** 2026-08-20

**Authors:** Joanna Reeves, Connor Thacker, Geoffrey Cunningham, Sam Hughes, Jennifer L. Davies

**Affiliations:** 1 School of Healthcare Sciences, Cardiff University, Cardiff, UK; 2 Faculty of Medicine, Health and Life Sciences, Swansea University, Swansea, UK; 3 Department of Clinical and Biomedical Sciences, Medical School Building, University of Exeter, Exeter, UK

**Keywords:** Nociceptive flexion reflex, Conditioned pain modulation, Lower-limb kinematics, Gait phase, Foot sole stimulation, Reflex modulation, Reflex response electromyography

## Abstract

Tonic pain inhibits the nociceptive withdrawal reflex (NWR). NWR magnitude varies across the gait cycle, but it is unknown whether tonic pain affects this phase-dependent modulation of the NWR during walking. Sixteen healthy participants (mean ± SD age: 29 ± 9 years, 3 males) walked on a treadmill with and without tonic pain applied to the left arm. Lower-limb kinematics and electromyography (EMG) from bilateral biceps femoris, vastus lateralis, soleus and tibialis anterior were recorded. At predefined points in the gait cycle, a brief electrical stimulus was applied to the arch of the right foot to elicit an NWR. Reflex responses were calculated from the EMG as a percentage difference to the EMG present without any stimulation. There was a significant effect of gait phase on reflex response in all muscles except ipsilateral biceps femoris. When accounting for the intensity of tonic pain, there was a 0.64 decrease in normalised reflex response in the contralateral tibialis anterior with tonic pain versus control (*p* = 0.009). There were no other effects of tonic pain on reflex response (*p* > 0.05). There was an increase in knee flexion on the stimulated leg when it was unloading, however there was large variability in the kinematics. Stimulation during loading response saw around a 3° increase (*p* = 0.007) in dorsiflexion in the contralateral leg. Our findings suggest that tonic pain does not influence the modulation of the NWR during walking. We propose that the need to maintain balance overrides any effects of endogenous pain modulation.

## Introduction

The nociceptive withdrawal reflex (NWR) is a polysynaptic spinal, protective reflex that is triggered by activation of A-delta (type III) sensory fibres (Wiesenfeld-Hallin et al. 1984). This activation produces a coordinated pattern of muscle activity that rapidly withdraws the stimulated area from a noxious, potentially tissue-damaging, source. The magnitude of the NWR can be quantified through the magnitude of changes in muscle activation and kinematics (Duysens et al. 1990, 1992; Spaich et al. 2004, 2006, 2009; Richard et al. 2015). The size of the NWR response is dependent on many factors, including the stimulus location (Andersen et al. 1999) and the position of the limb (Hagbarth and Finer 1963). The NWR can also be modulated by the central nervous system through ‘top-down modulation’, which refers to the regulatory processes by which higher brain centres (such as the cortex), influence the processing and perception of nociceptive signals at lower levels of the central nervous system (such as the spinal cord) (Fields et al. 1983; Basbaum and Fields 1984). Top-down modulation of spinal nociception is thought to underlie the concept of conditioned pain modulation (CPM), in which pain from an initial (test) stimulus is affected (either facilitated or inhibited) by a conditioning painful stimulus elsewhere on the body (Ramaswamy and Wodehouse 2021). As would be expected, these conditioning stimuli inhibit the NWR measured when participants are supine or sitting (Willer et al. 1984, 1989; France and Suchowiecki 1999; Terkelsen et al. 2001; Serrao et al. 2004; Biurrun Manresa et al. 2014; Jure et al. 2019).

The magnitude of the NWR observed in the lower limb also varies throughout the gait cycle (loading response, mid-stance, terminal stance, pre-swing and mid-swing) (Crenna and Frigo 1984; Duysens et al. 1992; Spaich et al. 2004, 2006; Richard et al. 2015). This is likely due to a combination of afferent and efferent information and possibly spinal motor programs (Spaich et al. 2004), and means that the consequence of the NWR is always protective – withdrawing the stimulated area from potential tissue damage while also maintaining balance and maintaining forward progression (Crenna and Frigo 1984; Spaich et al. 2004). Analysing electromyography (EMG) and kinematics simultaneously at specific phases of the gait cycle allows us to understand the functional relevance of the magnitude of the NWR. For instance, knee flexion has been shown to be greater when stimulation occurred in pre-swing and swing compared to heel-contact and midstance (Spaich et al. 2004). Excessive knee flexion at heel-contact when the knee is extended and loading begins or in a period of single support like midstance could lead to limb collapse and/or a fall, whereas greater knee flexion in pre-swing or swing would be of less consequence.

While there is clear evidence that tonic pain delivered to distant body regions can activate endogenous top-down modulatory mechanisms that can inhibit the NWR evaluated in static conditions, and clear evidence of phase-specific NWR magnitude during walking, it is not known if a tonic pain conditioning stimulus impacts the phase-dependent modulation of the NWR during walking. If it does, this could have critical implications for the ability (a) to withdraw the limb from a potentially tissue-damaging source and (b) to maintain balance while walking when the NWR is activated. Understanding the impact of ongoing pain on the phase-dependent modulation of the NWR during walking has important implications for populations in chronic pain. We expected NWR responses to vary across the gait cycle during pain-free walking, as previously observed. However, whether the same phase-dependent modulation would persist, be reduced, be enhanced, or be absent during tonic pain was treated as exploratory.

### Research question

Does tonic pain impact the modulation of the NWR through the gait cycle in healthy individuals?

## Methods

Sixteen healthy participants (mean ± SD age: 29 ± 9 years, 3 males) participated in this study. Ethical approval was obtained from Cardiff University School of Healthcare Sciences Research Ethics Committee (REC1181) and all participants provided informed consent. Individuals were eligible to take part if they were between 18 and 65 years of age, able to understand English and able to walk on a treadmill for 10 min at a time. Exclusion criteria were: neurological or musculoskeletal conditions affecting balance, walking or muscle activity; medical conditions prohibiting delivery of electrocutaneous stimulation to the foot or affecting pain processing or circulation (including uncontrolled high blood pressure of ≥ 140/90 mm Hg at the start of the session); taking any medications that could affect pain processing; acute illness; psychiatric disorders; substance disorders; current consumption of products containing nicotine; allergy to adhesive; pregnancy and body mass ≥ 135 kg (due to the limit of the treadmill safety harness).

### Procedure

Height and body mass were recorded with a stadiometer and scales, respectively. Blood pressure was measured twice with participant seated, using an electronic blood pressure monitor. The higher of the two readings was retained. This served as a screening tool for exclusion and to establish the magnitude to inflate the manual blood pressure cuff during the tonic pain walking condition (details provided below).

#### Placement of EMG electrodes

Recording surface electrodes (Avanti, Delsys Trigno; Delsys, Natick, MA, USA) were used to record the activity of the tibialis anterior (TA), soleus (SOL), vastus lateralis (VL) and biceps femoris (BF) muscles of both legs. Electrodes were positioned according to The European project Surface EMG for non-invasive assessment of muscles (SENIAM) guidelines (Hermens et al. 2000). The skin in the region of electrode placement was prepared by shaving the area (if required), applying an abrasive paste and wiping the skin with an alcohol wipe. Electrodes were affixed to the skin using a double-sided adhesive sticker (Trigno adhesive interface; Delsys) with a cohesive bandage wrapped around the sensor and limb for additional security. Correct electrode placement was confirmed by asking the participant to perform movements and observing the raw signal, with placement adjusted if necessary.

Stimulation surface electrodes (Neuroline 715; Ambu, Baltorpbakken, Denmark) were used to elicit the NWR. The participant was asked to remove the shoe and sock on their dominant (right) foot to allow placement of the self-adhesive stimulation electrodes. One electrode (cathode) was placed on the arch of the foot and another (anode) on the dorsum of the foot. The arch was chosen as the stimulation site because this has been shown to generate a greater magnitude of NWR response than other sites on the plantar surface of the foot (Andersen et al. 1999; Spaich et al. 2004). Once placed, the participant was asked to carefully replace the sock and shoe, and the connecting wires were fed out the medial side of the shoe and taped at the ankle to ensure they were secure.

#### Familiarisation with the NWR and establishing NWR threshold

The NWR was elicited by a train of five constant current rectangular pulses, each lasting 2 ms and delivered with an inter-stimulus interval of 5 ms, giving a total stimulus duration of 22 ms. This is perceived as a single stimulus in the arch of the foot. The stimuli were delivered by a DS7A constant current stimulator (Digitimer, Welwyn Garden City, UK) connected to a DG2A Train Delay Generator (Digitimer). The DS7A is a general-purpose electrical nerve or muscle stimulator for human stimulation and is medically CE marked in the UK/EU by the Medical Device Directive. The DG2A is designed to work with the DS7A when a train of stimuli is required. This stimulation paradigm has been used during walking in healthy and patient populations (Spaich et al. 2004, 2006). A similar protocol (a train of 5 rectangular pulses of 1 ms over a period of 20 ms) has also been used in walking but with stimulation of the sural nerve (Duysens et al. 1990, 1992).

The participant was familiarised with the stimulation. Stimulation was first delivered at a low intensity, below perceptual threshold. The participant was asked if they felt anything, and if they were happy to continue. If they were happy, the intensity was increased and the stimulation repeated. This continued until the participant felt the stimulation – typically experienced as a mild tingling. At this point EMG data were sampled at 2000 Hz in Vicon Nexus software (Vicon, Oxford, UK). The DS7A outputs a TTL-compatible signal that was connected to a Trigno Analog Adapter (Delsys) and synchronously sampled at 2000 Hz in Vicon Nexus software (Vicon). This signal indicates the onset of each stimulation. The stimulation intensity was manipulated as described below, and the muscle responses to all subsequent stimulation were monitored, to evaluate when an NWR occurred.

**Fig. 1:**
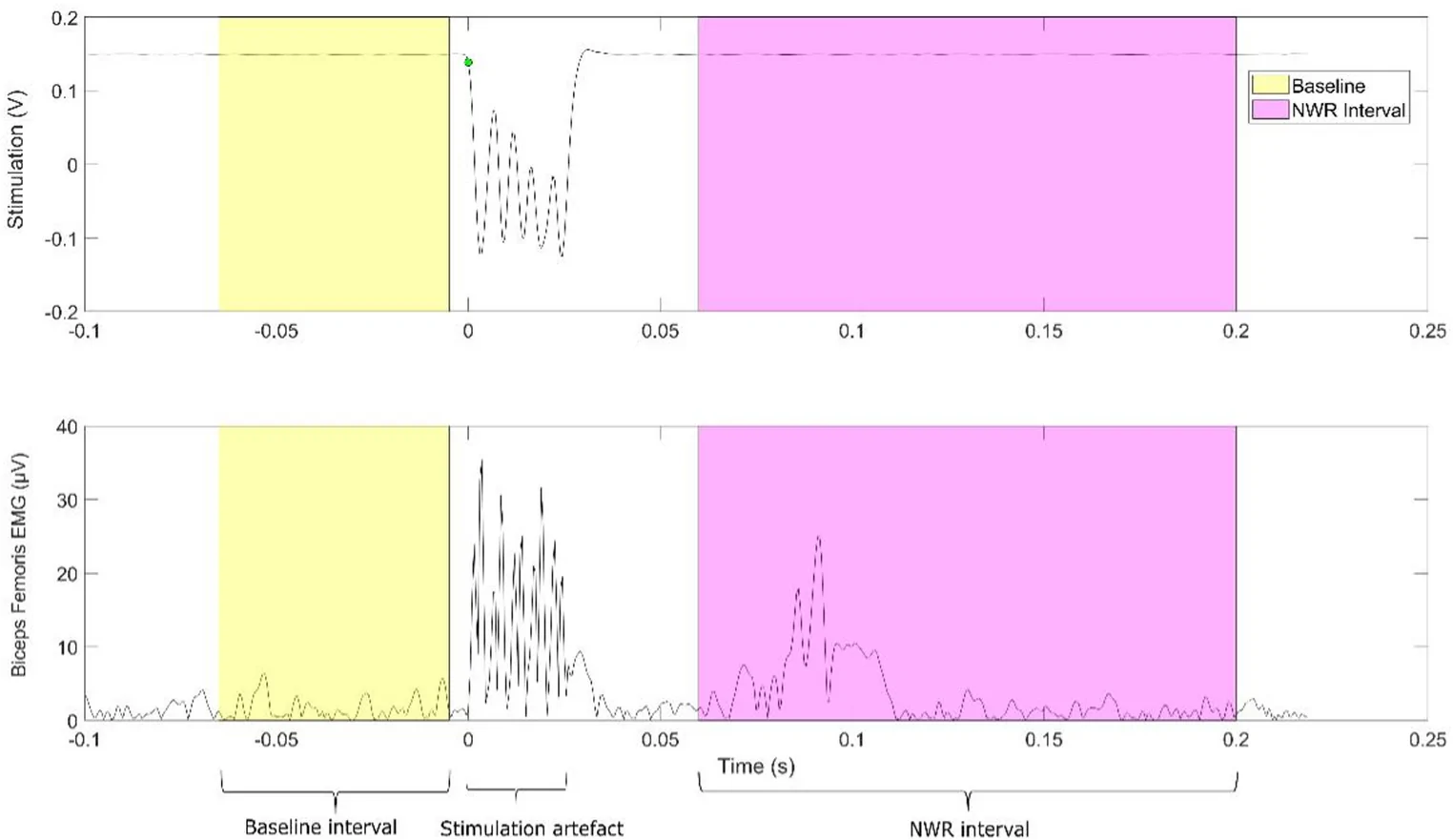
Threshold testing to identify a reflex response. Top panel shows the stimulus channel. Bottom panel shows the EMG recorded from the biceps femoris. Stimulus was delivered at time zero (green circle). A response was established if the interval peak z-score was ≥ 10. The z-score was calculated as the difference between the peak amplitude in the interval 60–200 ms post stimulation (pink box) and the mean baseline amplitude (yellow box), divided by the standard deviation of the baseline amplitude

To determine the stimulation intensity necessary to evoke a reflex response, the intensity was manipulated using a standardised staircase procedure. First, stimuli were administered with increasing intensity using steps of 2 mA until a reflex was observed. The reflex was defined as an interval peak *z*-score greater than 10 in TA and/or BF EMG, with a visible response, calculated as the mean rectified EMG amplitude in the 60–200 ms post-stimulation interval minus the mean rectified baseline EMG amplitude in the 120 ms pre-stimulation interval, divided by the standard deviation of the baseline EMG amplitude (Rhudy and France 2007; France et al. 2009). Reflex responses were confirmed by visual inspection (Fig. 1) using a custom-made MATLAB script (version 2017a, Mathworks Inc, MA, USA). Once a reflex was observed, the intensity was decreased in steps of 1 mA until no reflex was observed. This staircase method to obtain NWR threshold has been used previously (Jure et al. 2019).

Threshold detection was performed with the participant standing, with the leg that was not being stimulated on a solid wooden box that was ~ 25 cm high and the leg that was being stimulated hanging free (Rice et al. 2017). As such, the ipsilateral hip was in a neutral position, the ipsilateral knee was extended and the ipsilateral ankle in plantar flexion. Participants were able to hold on to a support if they became unbalanced and there was a spotter to prevent them from falling.

#### Placement of retroreflective markers for kinematic analysis

Retroreflective markers were placed on body landmarks according to two established models: The lower-limb Human Body Model 2 (HBM2; Motek Medical B.V., Netherlands) and the lower-limb Plug-in-Gait model (Vicon). Two models were necessary because different systems were used for real-time processing and offline processing of kinematic data. Real-time processing was performed within D-flow software as part of the Motek GRAIL system (Motek Medical B.V., Netherlands) and required the HBM2. Offline processing was performed with Vicon Nexus software and required the Plug-in-Gait model. The two models are similar, with many markers being used across both. The HBM2 model was therefore used only for real-time identification of heel strike. All kinematic outcomes reported were calculated using the Plug-in Gait marker model.

### Treadmill walking

#### Familiarisation

Participants were asked to walk on an instrumented treadmill (GRAIL, Motek Medical B.V., NL) while wearing a safety harness and with a spotter present. Once the participant was comfortable, the belt speed was increased until they declared they were at their comfortable walking speed. They then continued to walk at this speed for three minutes. This comfortable walking speed was recorded for subsequent walking trials, and average stride duration was calculated using a custom-made programme in D-flow software.

Kinematic data were sampled at 120 Hz in Vicon Nexus software. EMG data, ground reaction force data and the DS7A output signal were synchronously sampled at 2000 Hz in Vicon Nexus software. The position of the retroreflective markers was also streamed in real-time from Vicon Nexus software to D-flow software, where it was used for real-time monitoring of heel contact using change in the vertical velocity of the marker on the foot according to the method outlined by Zeni et al. (2008).

#### Walking with stimulation to elicit NWR

After the familiarisation period, participants performed trials where they walked at their comfortable walking speed with stimulation to elicit NWR under two conditions: (1) control; (2) tonic pain. The order of conditions was pseudorandomised across participants. Between trials, the participant was allowed to rest for as long as they wanted, before repeating the trial in the other condition (control/tonic pain).

At the start of the control trial, participants walked for 30-s without stimulation. After 30-s, stimulation was delivered once every 8–12 s (Rice et al. 2017) via the electrode on the arch of the foot as described above (five 2-ms constant current rectangular pulses with an inter-stimulus interval of 5 ms) at 1.2 times the NWR threshold detected in standing (Perrotta et al. 2017; Rice et al. 2017). Stimulation was delivered at one of five delays after heel strike, calculated to correspond to 0%, 20%, 40%, 60% or 80% of the average gait cycle duration during familiarisation, and continued in a pseudo-random manner until there had been five stimuli at each time point. The entire trial lasted 5–6 min.

During the tonic pain trial, pain caused by occlusion of the left upper arm was used as the conditioning stimulus. Before the start of the trial, a calibrated manual sphygmomanometer, also known as a pressure cuff, was placed around the upper arm contralateral to the stimulated foot. We chose a paradigm to provide a stimulus that was tailored to each individual and that we expected to induce moderate pain. Immediately prior to the start of the trial, the cuff was inflated to 1.5 times the participant’s diastolic blood pressure. Previous work has used inflation values of 1.5 systolic blood pressure (Willer et al. 1984), fixed values of 220–240 mmHg (France and Suchowiecki 1999, 2001; Campbell et al. 2008) or a target pain level of 3/10 on a verbal numerical rating scale (Coppieters et al. 2015). The gauge of the manual sphygmomanometer was clipped to the cuff or onto clothing and the participant was asked to hold the bulb in their hand during walking. The treadmill was then started, and once it reached the target speed, the stimulation started. The pressure cuff was inflated for a maximum of 5 min. Participants were asked to monitor the gauge of the cuff to reinflate if pressure dropped during walking. The participant was asked to verbally rate their pain on a numerical pain rating scale (NPRS) immediately after cuff inflation and then every minute during the five minutes of walking. The NPRS is an 11-point scale (0–10) for self-report of pain that was visually displayed to the participant and was anchored at zero with “no pain” and 10 at “the worst pain imaginable”. Participants were told they could release some pressure if the pain reached 7/10 (Riquelme-Aguado et al. 2024). The appropriate cuff size was used for each participant and cuffs were wiped clean between participants.

### Data analysis

**Fig. 2:**
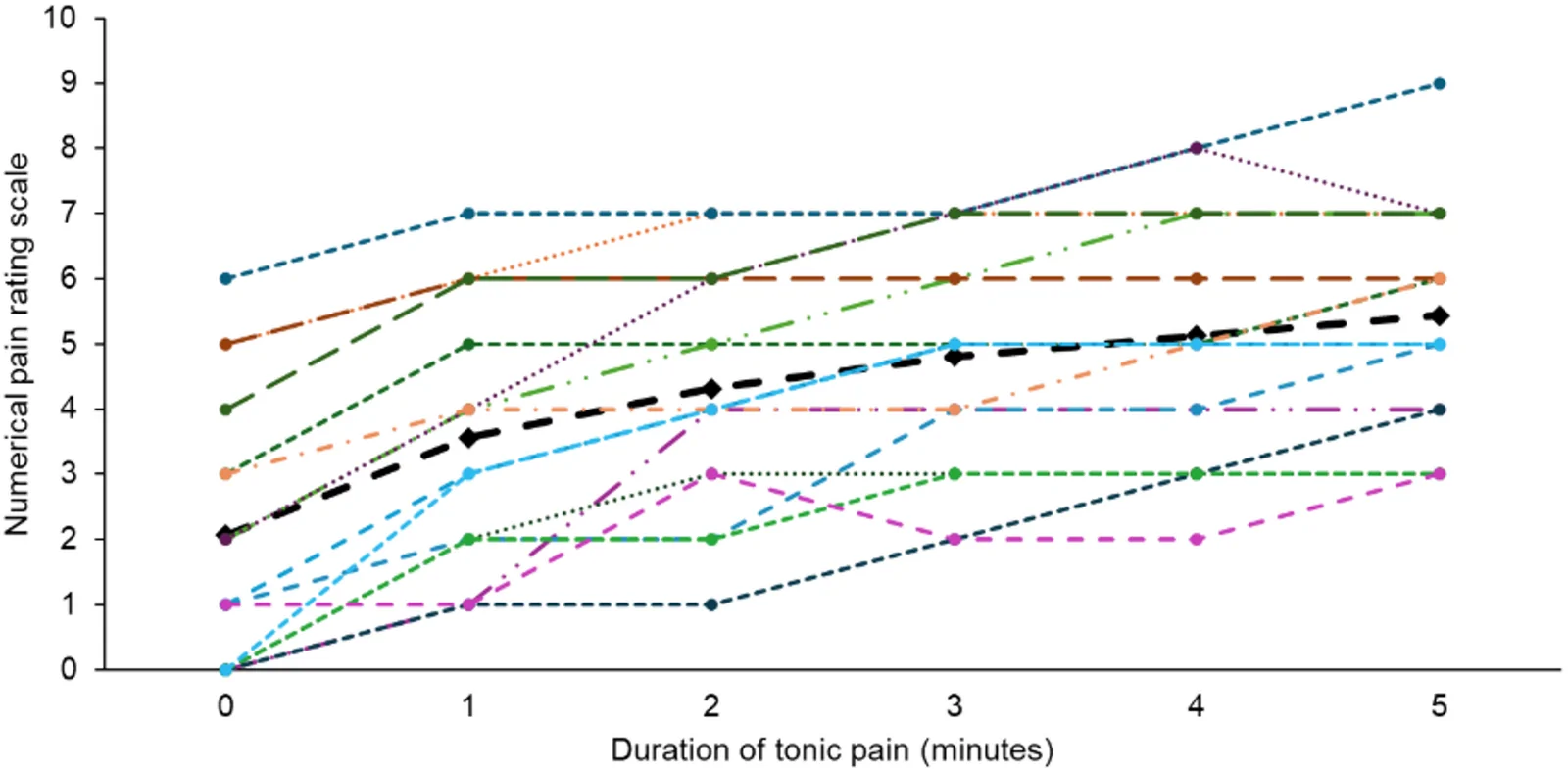
Pain ratings over time (*n* = 16) in the tonic pain trial. Coloured lines represent individual participants. Dashed black line represents the mean

Offline data analysis and visualisation was performed in MATLAB (version R2024b, Mathworks Inc), Microsoft Excel and Inkscape (version 1.4.2, Inkscape Developers, 2025). In control trials, the first stimulus was excluded from analysis to minimise non-steady-state effects associated with initial stimulus exposure. In tonic pain trials, all stimuli in the first minute of walking were excluded from analysis due to low NPRS ratings (Fig. 2).

#### Stimulus timing

The timing of stimulation during the trial was based on the average stride time during familiarisation and targeted at every 20% of the gait cycle. During offline analysis, the actual timing of each stimulus within the stride was calculated and categorised as occurring during loading response, mid-stance, terminal stance, pre-swing or mid-swing (Perry 1992). Stimulation onset was determined from the DS7A output signal using a negative threshold defined as baseline mean minus 4.5 times the baseline standard deviation, where baseline was the period 50–125 ms before the minima of the first pulse.

#### Gait phases

Vertical ground reaction force data were filtered using a second order lowpass filter with a cutoff of 10 Hz. Heel contact and toe-off of both feet were determined using a 20 N threshold in vertical ground reaction force (Hendershot et al. 2016). Loading response corresponds to the first period of double support in the gait cycle (Whittle 1991) and was defined as the period from right heel contact before left toe-off. Mid-stance was defined as 10–30% of the gait cycle, inclusive. Terminal stance was defined as > 30% and ≤ 50% of the gait cycle. Pre-swing corresponds to second double support and was defined as > 50% of the cycle, and greater than or equal to left heel contact and less than right toe-off (Whittle 1991). Mid-swing, which ends with the tibia vertical and the foot parallel with the floor (Liu et al. 2014; Leal-Junior and Frizera-Neto 2022) is often regarded as being between 73 and 87% of the gait cycle (Leal-Junior and Frizera-Neto 2022). However, it has been shown that mid-swing can end at up to 93% of the gait cycle (Liu et al. 2014). From the perspective of the NWR function, the key aspect was that the foot was unloaded, therefore we chose the broad classification of 73–93% of the gait cycle.

#### Reflex response EMG

EMG signals were bandpass filtered at 5–500 Hz (second order Butterworth), rectified, and root mean square (RMS) amplitude was calculated using a 25-ms window. RMS EMG was segmented into gait cycles (defined by heel contact). Gait cycles immediately post stimulation were removed. A random sample of 30 cycles from the remaining non-stimulation cycles were selected. From those 30 cycles, those with non-representative EMG profiles for each muscle were removed via visual inspection, leaving a minimum of seven cycles (mean ± standard deviation: 23 ± 10 cycles) per condition per participant. This data cleaning step was applied only to the averaged background profiles used to describe the typical gait cycle.

The time frame of interest post stimulus for TA, BF and VL on the stimulated leg and all muscles on the contralateral leg was 60–220 ms (Richard et al. 2015). For the SOL on the stimulated leg the time frame of interest was split into two: a short reflex loop (latency 60–120 ms) and a long reflex loop (latency 120–200 ms) (Spaich et al. 2004). For each of the five defined phases of the gait cycle, the average delay (ms) between heel strike and stimulus onset was calculated for each participant. This delay was used to define a corresponding period in the EMG profile from the unstimulated cycles (heel contact + average delay + window of interest). The reflex response was thus calculated as the average EMG during the period of interest in the stimulated trial minus the EMG in the corresponding period in the unstimulated trials. For each participant, RMS EMG for each gait cycle was time normalised to 101 points, a grand average profile across the gait cycle (101 points) was calculated across all non-stimulated cycles, and the grand mean of all 101 data points was calculated from this average profile. In both the control and the tonic pain conditions, the reflex response was normalised to this grand mean (single value) of EMG across the whole gait cycle in the unstimulated strides from the control walking trial (Richard et al. 2015). Signals were visually inspected during the calculation of reflex response to check for artefacts in the data. This was applied only to exclude trials with clear non-physiological artefact and was not used to exclude trials with ‘non-representative’ EMG (as was done in the creation of the EMG profiles used to describe the typical gait cycle). No trials were excluded at this step.

#### Kinematics

Kinematics were processed in Vicon Nexus software using the Plug-in Gait model. Gaps in trajectories were automatically filled with the Fill gaps pipeline (Woltring). Hip, knee and ankle angles in the sagittal plane were extracted for both legs. Joint angle time series were filtered using a 4th order lowpass Butterworth filter with a frequency cutoff of 6 Hz and for each gait cycle were time normalised to 101 points. The same non-stimulation trials for the EMG were used in the kinematic analysis, further non-representative trials were removed via visual inspection if necessary, leaving a minimum of four cycles (mean ± standard deviation: 15 ± 5 cycles) per condition per participant. This data cleaning step was applied only to the averaged background profiles used to describe the typical gait cycle. A mean for each condition was calculated across 201 points, corresponding to the gait cycle in which the stimulation occurred and the subsequent cycle, to be able to analyse whether an effect of stimulation carried over into the next gait cycle.

#### Statistics

Statistical analysis was performed using custom scripts in R (2025.05.1, Build 513, Posit Software, PBC, Boston, USA). For reflex responses, data points were identified as outliers and excluded if they were 1.5*interquartile range below or above the first or third quartile, respectively. This resulted in exclusion of 66 of 1231 responses (5.4%). Linear mixed models were fitted using the lme4 package (Version 1.1-37, Bates et al. 2015), Models included fixed effects of gait phase and condition (tonic pain vs. control) and their interactions. Sex and pain level (NPRS score) at the end of the tonic pain condition were included as covariates. To account for repeated observations within individuals and interindividual differences in baseline reflex responsiveness, participant was included as a random intercept. Sex was explored as a factor in the model as the evidence for its influence on the NWR is inconclusive (Linde et al. 2021). An analysis of variance (ANOVA) was used to compare models and the Akaike information criterion (AIC) used to compare model fit where necessary. Estimated marginal means and pairwise comparisons between gait phases were calculated with a Bonferroni correction using the package emmeans (Version 1.11.2, Lenth 2023). Models were inspected for departures from normality by visual inspection of Q-Q plots and histograms of the residuals of the model fit. Level of significance was set at α = 0.05. Data in figures are reported as mean ± standard deviation and range.

Kinematics were analysed using statistical parametric mapping (SPM) (Pataky 2010). For each joint (hip, knee, ankle), side (right/left) and phase of the gait cycle in which a stimulation occurred (loading response, midstance, terminal stance, pre swing, swing) a two-way repeated-measures ANOVA was run, with main effects of condition (tonic pain, control) and NWR stimulation (yes, no) and a region of interest (ROI) including the gait phase plus an additional 30% of the cycle after the phase. An ROI was used to augment statistical power around the time we would expect an effect of the stimulation (Pataky et al. 2016). Level of significance was set at α = 0.05. In contrast to the EMG analysis, a significant main effect of condition in the kinematic analysis indicates that the joint angle differed between the control and tonic pain conditions, rather than indicating a difference in the response to NWR stimulation. The effect of tonic pain on the kinematic response to NWR stimulation is indicated by the condition*stimulation interaction. When a main effect or interaction effect was identified, a post hoc analysis was performed as a paired t-test with the corresponding ROI and a Bonferroni correction so that α was divided by the number of comparisons of interest. For post hoc comparisons the difference between signals for each participant was calculated and a mean and standard deviation of the differences was subsequently calculated. A continuous Cohen’s d effect size was calculated along with each post hoc comparison. It has been suggested that thresholds for interpretation of continuous effect sizes should be substantially higher than with discrete data (Pataky et al. 2026).

## Results

### Reflex response (EMG)

The average stimulus intensity used during the walking trials was 10.5 ± 3.2 mA (range 4.8–14.4 mA). The average cuff inflation during the tonic pain walking trials was 106 ± 13 mmHg (range 83–126 mmHg). Average NPRS at the end of the tonic pain condition was 5/10 ± 2/10 (range 3/10–9/10). NPRS ratings over time in the tonic pain condition are shown in Fig. 2.

**Fig. 3:**
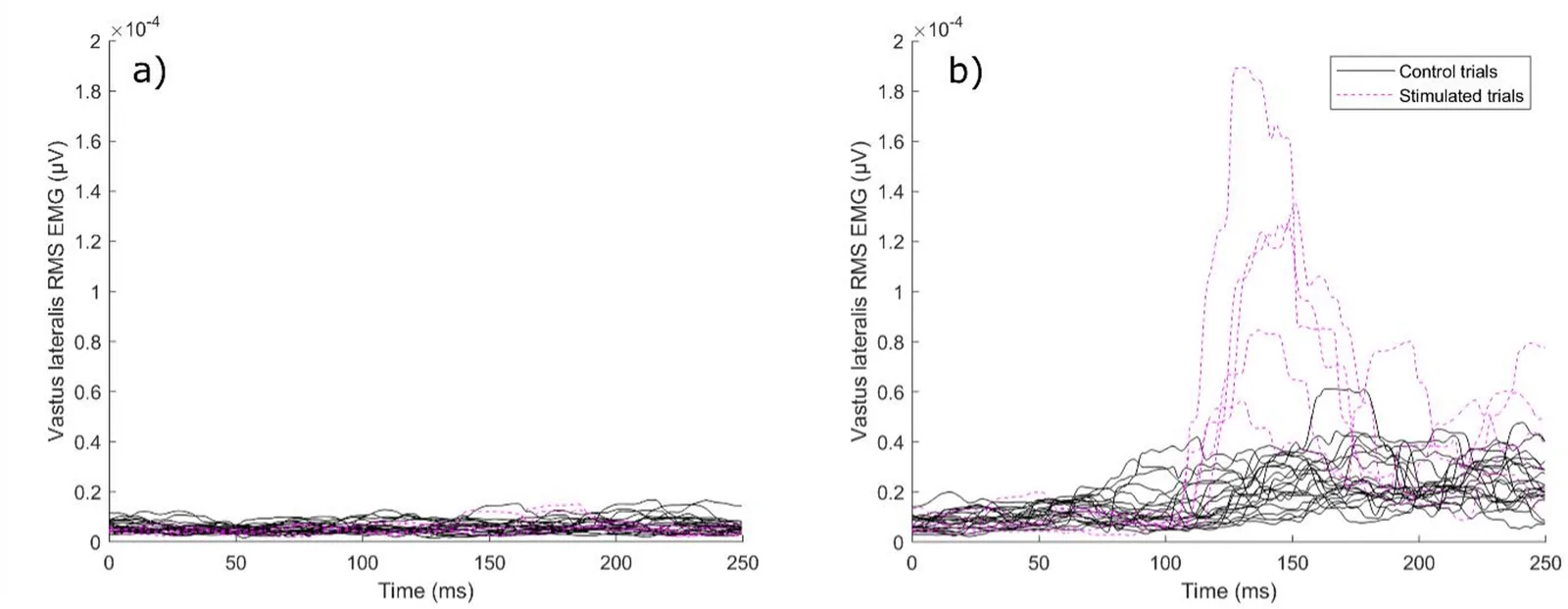
Reflex response in the vastus lateralis muscle of the stimulated leg during a control trial from one example participant in **a** terminal stance and **b** mid-swing phase. The black lines represent EMG during non-stimulated trials and magenta dashed lines represent EMG during a stimulation trial. Time 0 indicates when the stimulus was delivered. There is a large reflex response (magenta dashed lines) evident in mid-swing (77% of gait cycle) that is absent in terminal stance (37% of gait cycle)

**Fig. 4:**
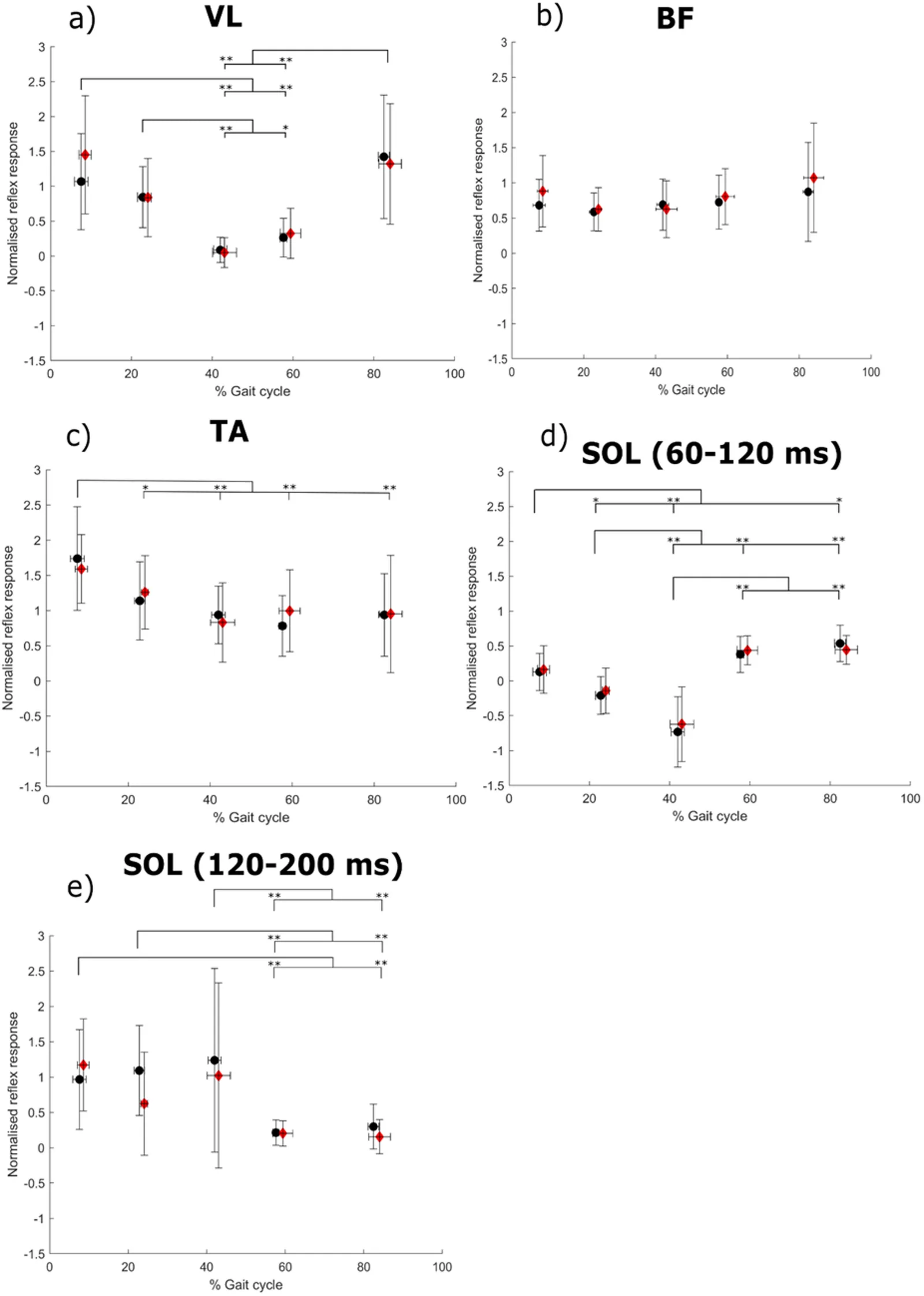
Mean relative reflex response in the leg ipsilateral to the stimulation across the gait cycle for: **a** vastus lateralis (*n* = 15); **b** biceps femoris (*n* = 14); **c** tibialis anterior (*n* = 16); **d** soleus short loop (60–120 ms post stimulation, *n* = 14); and **e** soleus long loop (120–200 ms post stimulation, *n* = 14). Dark blue circles represent control condition and red diamonds represent the tonic pain condition. Error bars represent the standard deviation. * denotes significant difference in phase (*p* < 0.05), ** denotes significant difference in phase (*p* < 0.001)

**Fig. 5:**
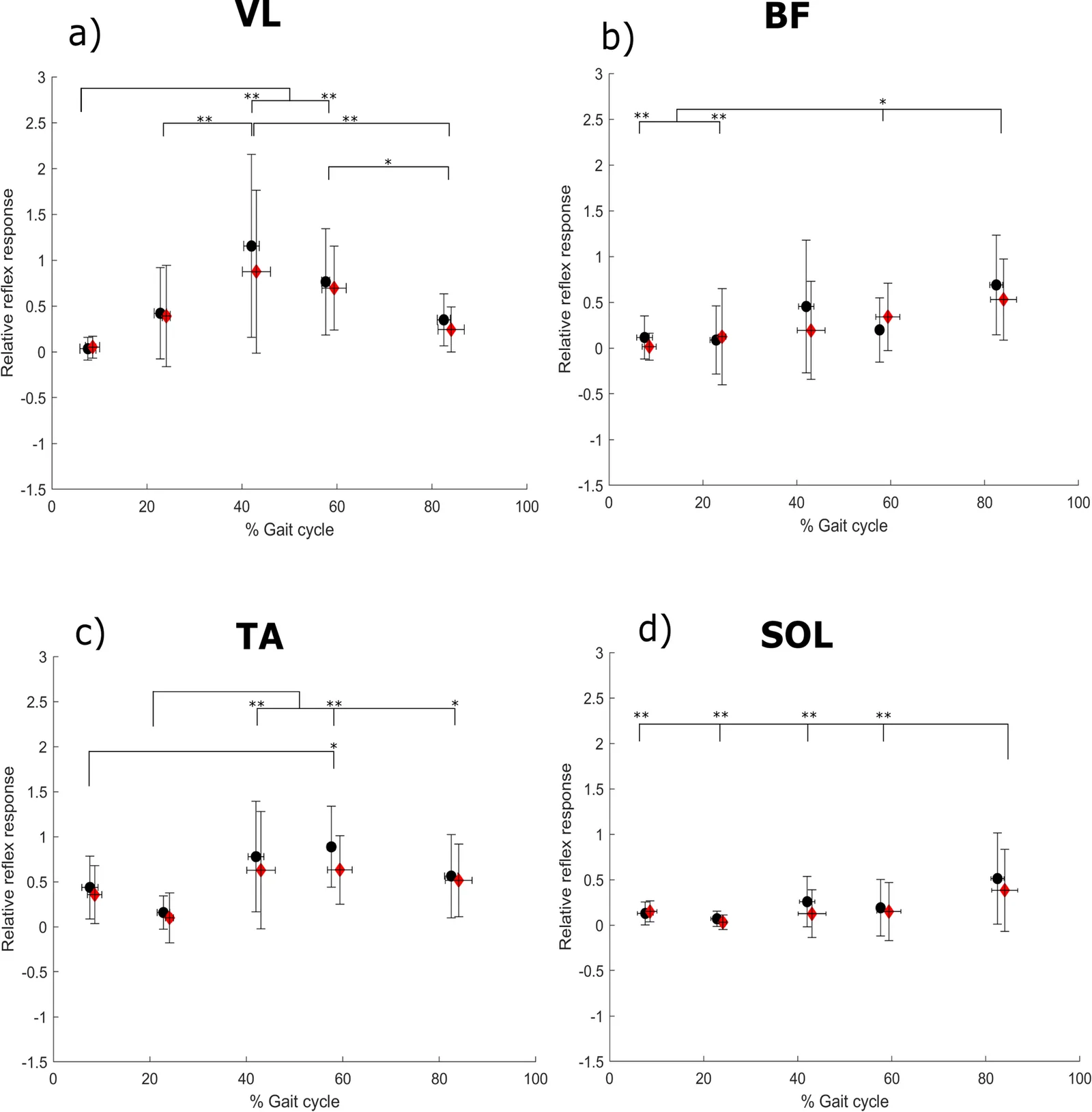
Mean relative reflex response in the leg contralateral to the stimulation across the gait cycle for: **a** vastus lateralis (*n* = 15); **b** biceps femoris (*n* = 14); **c** tibialis anterior (*n* = 16) and **d** soleus (*n* = 14). Dark blue circles represent control condition and red diamonds represent the tonic pain condition. Error bars represent the standard deviation. * denotes significant difference in phase (*p* < 0.05), ** denotes significant difference in phase (*p* < 0.001)

Technical issues meant there was usable EMG data in each leg for: vastus lateralis *n* = 15; biceps femoris *n* = 14; tibialis anterior *n* = 16 and soleus *n* = 14. Figure 3 shows example VL EMG during terminal stance and mid-swing for stimulated and non-stimulated gait cycles in the control (no pain) walking condition. Figures 4 and 5 show normalised reflex response across the gait cycle for both control and tonic pain conditions.

#### Fixed effects in the linear mixed model

For most muscles, tonic pain did not have an effect on reflex response (no effect of condition; all *p* > 0.05; Figs. 4 and 5). The sex of the participant did not have a significant effect on reflex response, so was removed as a fixed effect from the linear mixed models. NPRS score at the end of the tonic pain condition did not have a significant effect on reflex response except for in TA in the leg contralateral to the stimulation (*p* = 0.03). With NPRS score included in the linear mixed model the effect of condition on reflex response in the contralateral TA was significant (a decrease of 0.64 in normalised reflex response with respect to control, *p* = 0.009). The AIC of the model including NPRS was 232.92 compared with 231.29 not including NPRS and the difference between the models was not significant (*p* = 0.54). In the interest of parsimony (keeping a model as simple as possible while still explaining the data adequately), the fixed effect of NPRS was removed from all models prior to the calculation of pairwise comparisons between gait phases. Without NPRS score included in the linear mixed model there was not a significant effect of condition on reflex response in the TA in the leg contralateral to the stimulation (*p* = 0.11). There was a significant effect of gait phase on reflex response in all muscles except for BF on the leg ipsilateral to the stimulation (Fig. 4b). Significant post hoc pairwise comparisons between gait phases are indicated in Figs. 4 and 5.

### Kinematics

**Fig. 6:**
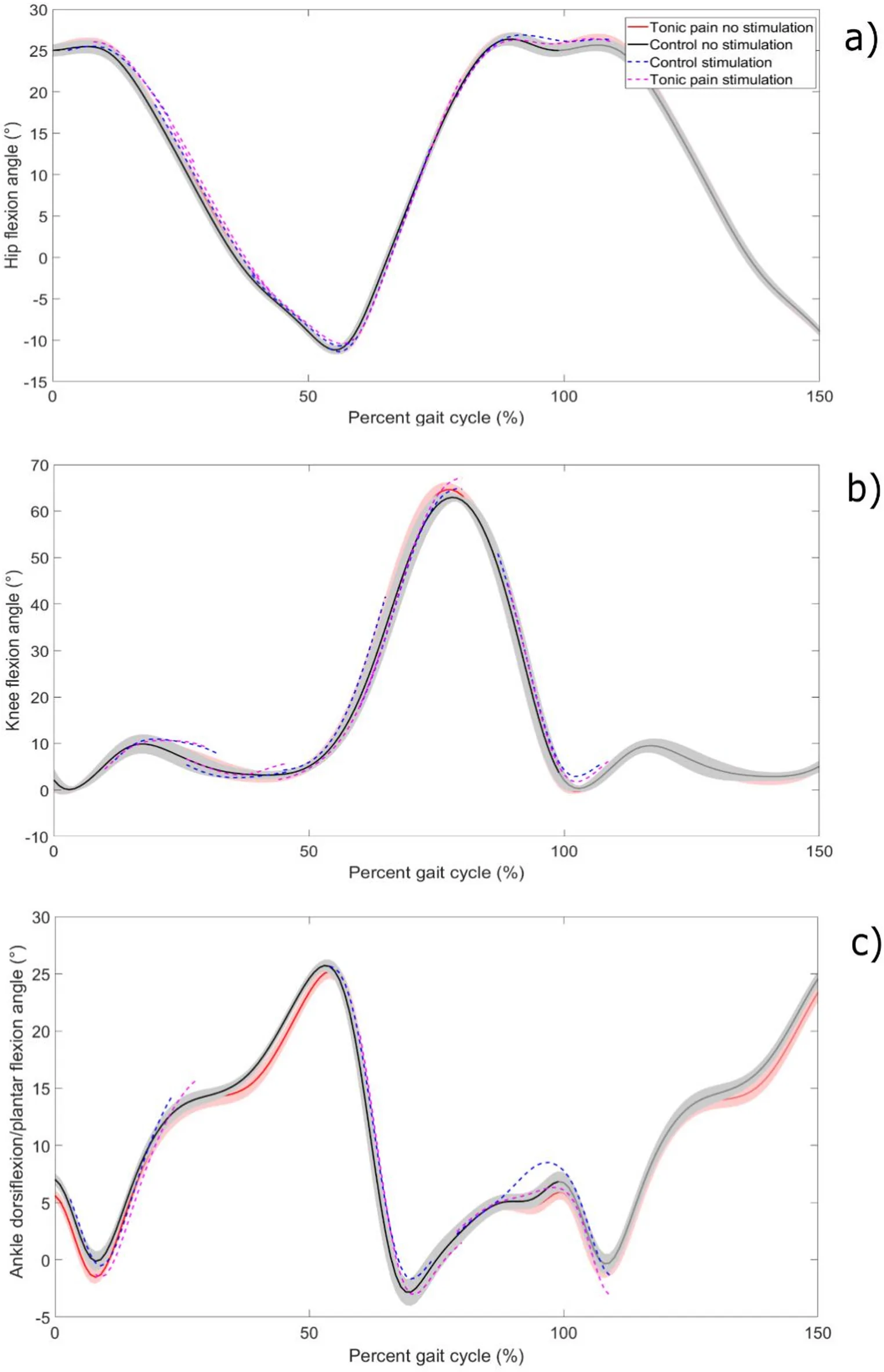
Example response to stimulation at the **a** hip, **b** knee and **c** ankle in the leg ipsilateral to the stimulation, normalised to the gait cycle (time zero is heel strike of the stimulated leg). Data are shown from the hip and ankle of one participant and the knee of another participant. Black lines with grey shading represent mean ± SD strides in the control condition without stimulation. Red lines with lighter red shading represent mean ± SD strides in the tonic pain condition without stimulation. Blue dashed lines represent strides in the control condition with stimulation and magenta dashed lines represent strides in the tonic pain condition with stimulation (both from average stimulation time plus 20% of the gait cycle). Stimulation shown for 0%, 40%, 60% and 80% to maintain visual clarity

Usable kinematic data were available for *n* = 10 participants. The mean responses to stimulation at each joint during the control and tonic pain conditions are shown in Fig. 6 (from the hip and ankle of one participant and the knee of another participant). The full pattern of SPM ANOVA results is summarised in Table 1. Overall, kinematic responses to stimulation were small and phase-dependent. Evidence that tonic pain altered the kinematic response to NWR stimulation was limited to condition × stimulation interactions for ipsilateral hip and knee angles during terminal stance, ipsilateral hip, knee and ankle angles during pre-swing, and contralateral hip angle during pre-swing. Main effects of stimulation were observed more widely, particularly during loading response, but these effects generally reflect small stimulation-evoked changes rather than modulation by tonic pain. Main effects of condition are reported separately, as these indicate differences in joint angle between the tonic pain and control conditions, rather than differences in the stimulation-evoked response.

**Table 1:** Summary of statistical parametric mapping repeated-measures analysis of variance p-values for lower-limb kinematics

	Loading response			Mid stance			Terminal stance			Pre swing			Swing		
	Cond	Stim	Cond× Stim	Cond	Stim	Cond× Stim	Cond	Stim	Cond× Stim	Cond	Stim	Cond× Stim	Cond	Stim	Cond× Stim
Ipsilateral hip	n.s.	n.s.	n.s.	n.s.	n.s.	n.s.	n.s.	n.s.	0.044	n.s.	0.046	0.040	n.s.	0.044	n.s.
Ipsilateral knee	0.025	0.0499	n.s.	n.s.	n.s.	n.s.	0.019	n.s.	0.047	0.049	n.s.	0.037	n.s.	n.s.	n.s.
Ipsilateral ankle	0.046	0.041	n.s.	n.s.	n.s.	n.s.	n.s.	n.s.	n.s.	n.s.	0.017	0.036	n.s.	0.050	n.s.
Contralateral hip	n.s.	n.s.	n.s.	n.s.	n.s.	n.s.	n.s.	n.s.	n.s.	n.s.	n.s.	0.050	n.s.	n.s.	n.s.
Contralateral knee	n.s.	0.045	n.s.	n.s.	n.s.	n.s.	n.s.	0.0495	n.s.	0.007	n.s.	n.s	n.s.	n.s.	n.s.
Contralateral ankle	n.s.	0.007	n.s.	n.s.	0.049	n.s.	n.s.	n.s.	n.s.	n.s.	n.s.	n.s	n.s.	n.s.	n.s.

Values are p-values for significant effects identified using statistical parametric mapping (SPM) repeated-measures analysis of variance (ANOVA) within the predefined region of interest. Separate two-way repeated-measures ANOVAs were conducted for each joint, limb and phase of the gait cycle, with factors of condition and stimulation. Cond = main effect of condition (tonic pain vs. control); Stim = main effect of NWR stimulation (stimulation vs. no stimulation); Cond × Stim = condition × stimulation interaction. Ipsilateral and contralateral refer to the limb relative to the stimulated side. n.s. indicates no significant effect. Main effects of condition indicate differences in joint angle between tonic pain and control conditions, whereas Cond × Stim interactions indicate whether tonic pain altered the kinematic response to stimulation.

#### Stimulation delivered during loading response

There was no condition × stimulation interaction for any ipsilateral or contralateral joint during loading response. For the ipsilateral limb, there were no main effects of condition or stimulation on hip angle. There were main effects of condition and stimulation on ipsilateral knee and ankle angles. For the knee, angle after stimulation in the tonic pain condition was significantly lower, indicating less flexion, than after stimulation in the control condition; however, this effect was very small with a maximum mean difference within the ROI of 1.1 ± 0.2° (*p* < 0.001, d ~ 0.1). For the ankle, post hoc comparisons were not significant.

**Fig. 7:**
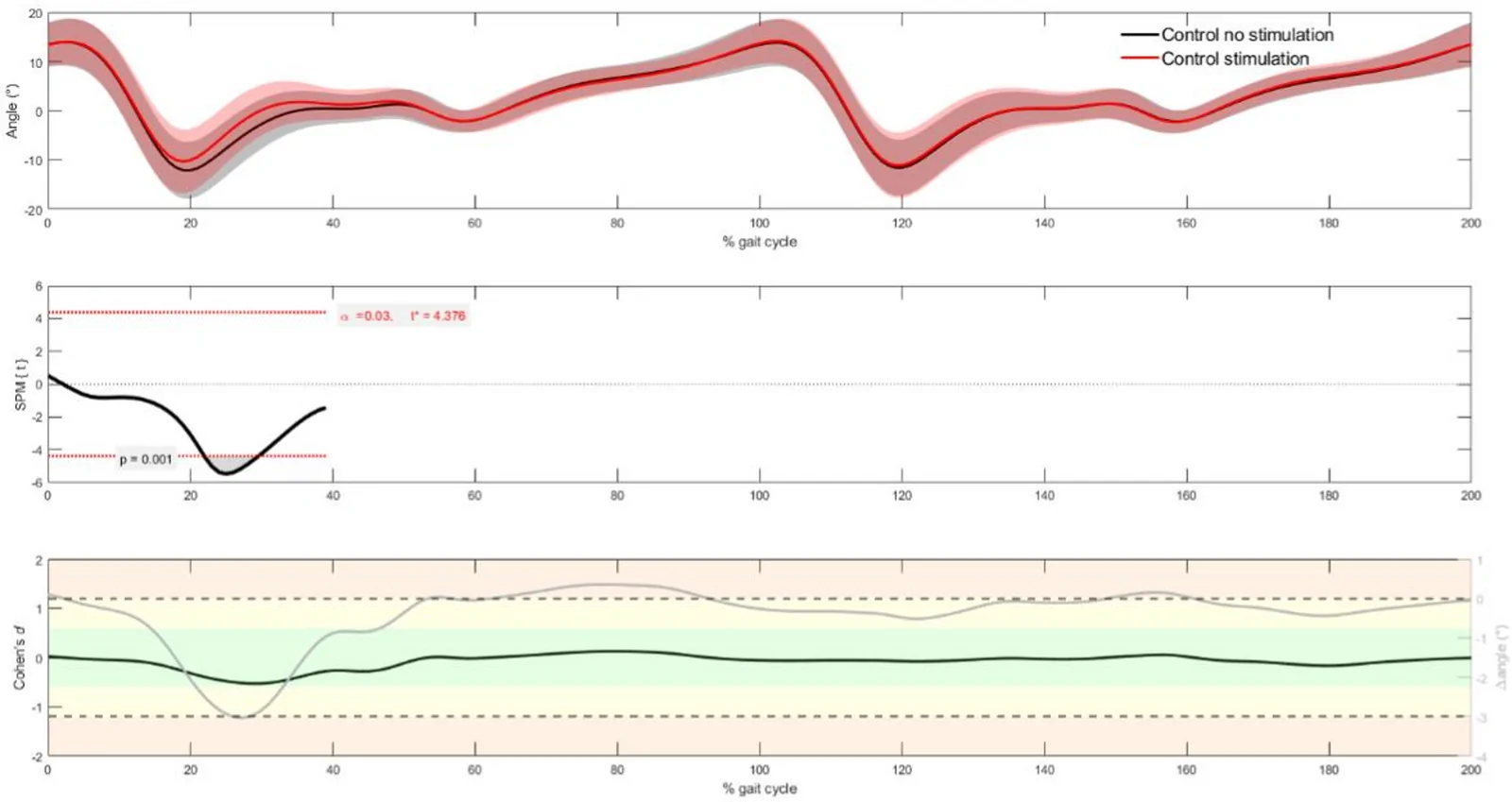
Kinematic response of the contralateral leg to stimulation of the ipsilateral leg during the ‘loading response’ phase of the gait cycle. Top panel shows plantarflexion/dorsiflexion angle of the strides in the control condition without stimulation (mean black line and standard deviation grey shading) and strides in the control condition when stimulation was delivered in the loading phase of the ipsilateral leg (mean red line and standard deviation pink shading). Middle panel shows SPM in the range of interest. Bottom panel shows Cohen’s d (black line) and difference between angles (grey line)

For the contralateral limb, there were no main effects of condition on hip, knee or ankle angle. There was no main effect of stimulation on contralateral hip angle. There were main effects of stimulation on contralateral knee and ankle angle. Post hoc comparisons for the knee were not significant. For the contralateral ankle, stimulation was associated with greater dorsiflexion in the control condition, with a maximum mean difference within the ROI of 2.8 ± 1.7° (*p* = 0.001, d = 0.52), and in the tonic pain condition, with a maximum mean difference of 3.4 ± 3.2° (*p* = 0.013, d = 0.75; Fig. 7).

#### Stimulation delivered during mid-stance

There was no condition × stimulation interaction for any ipsilateral or contralateral joint during mid-stance. There were also no main effects of condition or stimulation on ipsilateral hip, knee or ankle angle, or on contralateral hip or knee angle. There was a main effect of stimulation on contralateral ankle angle, but post hoc comparisons were not significant. There was no main effect of condition on contralateral ankle angle.

#### Stimulation delivered during terminal stance

During terminal stance, there were condition × stimulation interactions for ipsilateral hip and knee angle. For the ipsilateral hip, post hoc comparisons were not significant. For the ipsilateral knee, knee flexion after stimulation was greater in the control condition than in the tonic pain condition, with a maximum mean difference within the ROI of 6.8 ± 4.9°; however, this was not significant (d = 0.49). In the control condition, knee flexion after stimulation was greater than without stimulation, with a maximum mean difference of 5.7 ± 6.1°, but this was also not significant (d = 0.44). In the tonic pain condition, the maximum mean stimulation-related difference was 3.8 ± 3.6° and was not significant (d = 0.30).

There was also a main effect of condition on ipsilateral knee angle, but this should be interpreted cautiously given the significant condition × stimulation interaction. There was no main effect of condition on ipsilateral hip angle, no main effect of stimulation on ipsilateral hip or knee angle, and no interaction or main effects of condition or stimulation on ipsilateral ankle angle.

For the contralateral limb, there were no condition × stimulation interactions and no main effect of condition for any joint. There was a main effect of stimulation on contralateral knee angle, but post hoc comparisons were not significant. There were no main effects of stimulation on contralateral hip or ankle angle.

#### Stimulation delivered during pre-swing

The clearest evidence that tonic pain altered stimulation-evoked kinematic responses occurred during pre-swing, where there were condition × stimulation interactions for ipsilateral hip, knee and ankle angle.

For the ipsilateral hip, stimulation in the tonic pain condition increased hip flexion, with a maximum mean difference within the ROI of 2.1 ± 0.8° compared with no stimulation (*p* = 0.002, d = 0.20). In the control condition, the maximum mean stimulation-related difference was 3.8 ± 3.0°, but this was not significant (d = 0.34).

For the ipsilateral knee, knee flexion after stimulation was greater in the control condition than in the tonic pain condition, with a maximum mean difference within the ROI of 3.5 ± 10.7° (*p* = 0.002, d = 0.29). Within-condition comparisons of stimulation versus no stimulation were not significant in either the control condition (maximum mean difference 7.2 ± 12.8°, d = 0.54) or the tonic pain condition (maximum mean difference 6.0 ± 5.3°, d = 0.24).

For the ipsilateral ankle in the control condition, dorsiflexion after stimulation was greater than after no stimulation, with a maximum mean difference within the ROI of 3.3 ± 3.0° (*p* = 0.009, d = 0.44). In the tonic pain condition, dorsiflexion after stimulation was 2.5 ± 2.1° greater than after no stimulation, which was not significant (d = 0.34). The difference in angle after stimulation between control and tonic pain conditions was small, with a maximum mean difference of 1.3 ± 2.0° (being greater in the tonic pain condition).

There were also main effects of stimulation for ipsilateral hip and ankle angle, and a main effect of condition for ipsilateral knee angle. These should be interpreted cautiously given the significant condition × stimulation interactions.

For the contralateral limb, there was a condition × stimulation interaction for hip angle, but post hoc comparisons were not significant. There were no condition × stimulation interactions for contralateral knee or ankle angle. There was a main effect of condition for contralateral knee angle, but post hoc comparisons were not significant. There was no main effect of condition for contralateral hip or ankle angle, and no main effect of stimulation on any contralateral joint.

#### Stimulation delivered during swing

During swing, there were no condition × stimulation interactions and no main effects of condition for any ipsilateral or contralateral joint. There was a main effect of stimulation for ipsilateral hip angle. Hip flexion was greater after stimulation than after no stimulation in both the control condition (maximum mean difference within the ROI, 1.6 ± 1.0°, *p* = 0.010) and the tonic pain condition (1.5 ± 0.9°, *p* = 0.020). There was also a main effect of stimulation for ipsilateral ankle angle, but post hoc comparisons were not significant. There was no main effect of stimulation on ipsilateral knee angle or contralateral hip, knee or ankle angle.

## Discussion

This study demonstrated modulation of the NWR across the gait cycle but found little evidence that this modulation was altered by tonic pain in healthy individuals. At the level of EMG-defined reflex responses, tonic pain had little consistent effect, with the only condition-related finding being a decrease in normalised reflex response in the contralateral TA when accounting for level of pain experienced. In contrast, small condition × stimulation interactions were observed in the kinematic analysis, mainly during terminal stance and pre-swing. Overall, these findings suggest that, in healthy individuals, the phase-dependent modulation of reflex responses required for walking is largely preserved during tonic pain, although small changes in the resulting movement response may occur in specific phases of the gait cycle.

The lack of change in modulation of NWR across the gait cycle with tonic pain contrasts with previous studies showing reduced NWR amplitude and increased threshold during CPM paradigms when participants are supine or seated. For instance, the cold pressor test used to induce CPM significantly reduced the NWR response in the BF (Terkelsen et al. 2001) and rectus femoris (Jure et al. 2019) when participants were supine or seated, as well as increased the NWR threshold, increased the electrical pain detection threshold and decreased the pain intensity rating to suprathreshold electrical stimulation (Biurrun Manresa et al. 2014). Similarly, when forearm ischemia was used to induce CPM, NWR response in the BF measured when participants were seated was significantly reduced, and this persisted for at least five minutes post ischemia (France and Suchowiecki 1999).

It must be considered whether the tonic pain stimulus in the current study would have been sufficient to induce endogenous top-down pain modulatory pathways that are typically measured using CPM paradigms. However, average pain on the NPRS was 5/10 at the final minute, which is moderate pain and similar to other CPM protocols. Typical CPM protocols using ischemic pain on the arm as the conditioning stimulus have used 200 mmHg for up to 10 min or until NPRS of 6/10 is achieved (Ramaswamy and Wodehouse 2021), but others have used a target NPRS of as low as 3/10 and still observed CPM (Cathcart et al. 2009; Coppieters et al. 2015). Greater cuff pressure does not necessarily equate to greater pain levels; a cuff pressure of 240 mmHg resulted in average pain of only 62 ± 19 out of 100, but this was sufficient for a CPM effect (Lewis et al. 2012). When forearm ischemia was used to study the effect of CPM on NWR response, there was an average pain of 20/78 and 25/78 on the McGill Pain Questionnaire in men and women, respectively (France and Suchowiecki 1999). Visual inspection of individual reflex response plots did not suggest systematic differences between participants reporting lower than average pain intensity and those reporting average or higher pain. Additionally, adding NPRS scores to the linear mixed models did not generally improve model fit. Taken together, these findings suggest that the absence of an effect on NWR amplitude in the current study is unlikely to be explained by insufficient tonic pain stimulus. Indeed, the use of ischemic pain as a diffuse tonic pain conditioning stimulus has been shown to activate top-down endogenous pain modulatory mechanisms over the NWR during seated conditions (Terkelsen et al. 2001; Jure et al. 2019). However, these endogenous processes appear to have little or no effect over the NWR during the gait cycle.

Cognitive and attentional processes may influence spinal nociceptive processing through top-down modulation of descending control systems (e.g., Sprenger et al. 2012). In the present study, participants provided verbal pain ratings every 60 s during the tonic cuff stimulus. Although this intermittent rating procedure may have directed some attention towards the painful stimulus, it is unlikely to have provided a sustained or sufficiently strong cognitive manipulation to draw conclusions about top-down modulation. Studies that directly manipulate attentional engagement during tonic pain, for example by varying the frequency or nature of pain ratings, would be needed to determine the extent to which rating procedures influence spinal nociceptive processing.

The modulation of NWR across the gait cycle was similar to that previously reported with stimulation of the foot sole (Spaich et al. 2004, 2006, 2009; Richard et al. 2015). However, the magnitude of kinematic responses was smaller than in previous studies, particularly at the hip and knee. Previously, stimulating the arch of the foot to cause NWR resulted in increased knee flexion up to 20° after heel-off and swing and increased hip flexion up to 9° in swing (Spaich et al. 2004). This is compared to ~ 7° increased knee flexion in pre-swing in the current study and ~ 2° increased hip flexion in swing. A difference of 1–2° in flexion is arguably not meaningful given measurement error often 2–4° at the hip in the sagittal plane (McGinley et al. 2009). A lower magnitude of kinematic response in the present study could largely be due to a lower NWR stimulus intensity. In the study by Spaich et al. stimulus intensity was set as a factor of pain threshold with average intensity at the foot arch being 24.6 ± 11.5 mA, compared with 10.5 ± 3.2 mA in the present study. We were also limited to 10 participants for our kinematic analysis and had large inter-individual variability in knee kinematics, which may have reduced power and masked an effect of stimulation and/or pain. Nonetheless, our sample size was similar to previous studies of NWR ranging from 8 to 15 participants (Crenna and Frigo 1984; Duysens et al. 1990, 1992; Spaich et al. 2004; Spaich et al. 2009; Richard et al. 2015). We used a broad classification of mid-swing to try and retain as much data as possible, which meant that for a small number of participants the stimulation was delivered at a point that would traditionally be classed as terminal swing rather than mid-swing. However, visual inspection of the data did not suggest any systematic difference in these responses. A strength of the present study was the use of optical motion capture, which is more advanced than the goniometers used in previous work (Duysens et al. 1990, 1992; Spaich et al. 2004, 2006, 2009; Richard et al. 2015).

The present findings also suggest a possible difference between reflex amplitude in the recorded muscles and the resulting movement outcome. Tonic pain did not consistently alter EMG-defined reflex responses across the muscles recorded, whereas the kinematic analysis identified small, phase-dependent condition × stimulation interactions, particularly during terminal stance and pre-swing. One interpretation is that kinematic measures captured task-level consequences of tonic pain that were not evident in reflex responses from individual recorded muscles. Joint kinematics during walking reflect the integrated output of multiple muscles acting across multiple joints. EMG was recorded from a limited set of muscles, and changes in unrecorded muscles, altered muscle synergies, passive limb dynamics, or compensatory coordination strategies may have contributed to the observed kinematic effects. These effects may have been most apparent during terminal stance and pre-swing because these phases involve the transition from load-bearing and propulsion to limb unloading and swing initiation, requiring coordinated changes across the hip, knee and ankle. However, because the kinematic effects were small and phase-dependent, and not always supported by significant post hoc comparisons, this interpretation should be considered tentative.

To our knowledge the effect of NWR stimulation on contralateral ankle kinematics and TA reflex responses specifically during loading response reported in this study is a novel finding. Previous work has either only recorded kinematics and muscle activity from the ipsilateral leg (Spaich et al. 2004, 2006, 2009; Richard et al. 2015) or delivered stimuli at unspecified fixed intervals during gait and reported contralateral dorsiflexion in stance (Duysens et al. 1990, 1992). In the present study, when stimulation was delivered to the right foot during the loading phase of gait, there was a dorsiflexion response of around 3° in the left (contralateral) ankle, with a normalised reflex response in left TA of around 0.5. During the loading phase of the right foot, the left foot is plantarflexing and unloading in preparation for toe-off. Increased dorsiflexion at this time would slow this unloading, allowing more weight to be borne by the left foot while the right foot withdraws from a stimulus, facilitating maintenance of balance. There was no difference in kinematics of the left ankle when stimulation was delivered to the right foot in terminal-stance or pre-swing, but there was significantly increased left TA activity. This corresponds to the period approaching or during the second period of double support of the left leg, suggesting a possible role of increased weight bearing of the contralateral leg. This pattern was maintained in the presence of tonic pain.

The present study was conducted with young, healthy participants and it is not known if findings generalise to people living with chronic pain. This may be particularly important given evidence that chronic pain is associated with changes in NWR thresholds (Linde et al. 2021) and altered CPM (Pereira-Silva et al. 2025). At a behavioural level, chronic pain in older adults is linked to reduced gait speed (Seydi et al. 2025) and increased falls risk (Anbarasan and Merchant 2025) and chronic pain in clinical populations has been associated with poorer balance (Hirase et al. 2020) and balance confidence (Stubbs et al. 2014). Taken together, these findings suggest that nociceptive systems and the sensorimotor systems involved in balance and walking are altered in chronic pain. The modulation of the NWR throughout the gait cycle provides an ecologically valid context in which nociceptive processing must be dynamically integrated with motor control, but to our knowledge has never been investigated in people with chronic pain. Future studies aiming to understand whether phase-dependent modulation of the NWR is preserved, exaggerated, or disrupted in this population may provide important mechanistic insight into how pain-related changes in sensorimotor processing contribute to impaired gait and increased fall risk.

The present study was limited to analysis of activity of individual muscles. Previous work has used muscle synergy analysis to examine how tonic pain alters the net contribution of multiple muscles to the NWR during seated tasks (Jure et al. 2019). Such an approach could provide useful information about distributed changes in motor organisation during walking. However, synergy analysis requires careful consideration of muscle selection and normalisation. The consensus for experimental design in electromyography (CEDE) advised caution in calculating muscle synergies when normalised to the peak amplitude within a task (as done in the study by Jure et al.) as it can give unwarranted weighting to muscles with low or no activation (Besomi et al. 2020). A maximum voluntary contraction could be used for normalisation to calculate muscle synergies, but this is biased to muscles with high activation and ideally this would be optimised for each muscle. With EMG recordings for eight muscles in the present study, the recording of maximum voluntary contractions was considered unnecessarily time consuming and burdensome for the participants, especially given there is no agreement on the physiological meaning of synergy analysis and its interpretation (Besomi et al. 2020). Future studies with a larger set of recorded muscles and an optimised normalisation protocol could use synergy analysis to determine whether tonic pain alters coordinated multi-muscle responses to NWR stimulation during gait.

In addition, although participant-level variability was accounted for statistically by including a random intercept for participant, individual differences in nociceptive sensitivity, pain tolerance and perceived pain intensity over time may still have contributed to residual variability in reflex responses. The observed effects should therefore be interpreted as average group-level responses, rather than as effects expected to occur uniformly across all individuals.

In conclusion, we replicated phase-specific modulation of NWR amplitude across the gait cycle and found no evidence that tonic pain induced with a sphygmomanometer impacted this modulation at the level of EMG-defined reflex responses. Small, phase-dependent changes in kinematic responses were observed, but these effects were modest and should be interpreted cautiously. These findings suggest that, in healthy individuals, the need to modulate the NWR to maintain balance during walking overrides any effects of tonic pain.

## Data Availability

The data supporting the findings of this study are openly available in the Cardiff University Research Data Repository at https://doi.org/10.17035/cardiff.33256200.
